# Cognitive testing following transient ischaemic attack: A systematic review of clinical assessment tools

**DOI:** 10.1080/23311908.2023.2196005

**Published:** 2023-04-01

**Authors:** Alexander Hammant, Tamara Chithiramohan, Victoria Haunton, Lucy Beishon

**Affiliations:** 1 Department of Cardiovascular Sciences, University of Leicester, Leicester, UK; 2 Department of Cardiovascular Sciences, NIHR Leicester Biomedical Research Centre, British Heart Foundation Cardiovascular Research Centre, Glenfield Hospital, Leicester, UK

**Keywords:** Transient ischaemic attack (TIA), cognitive impairment, executive function, cognitive assessment tools, validation studies, Mini-mental state examination (MMSE), Montreal Cognitive Assessment (MoCA), Neuropsychological battery

## Abstract

Cognitive deficits are prevalent after transient ischaemic attack (TIA) and result in loss of function, poorer quality of life and increased risks of dependency and mortality. This systematic review aimed to synthesise the available evidence on cognitive assessment in TIA patients to determine the prevalence of cognitive deficits, and the optimal tests for cognitive assessment. Medline, Embase, PsychINFO and CINAHL databases were searched for relevant articles. Articles were screened by title and abstract. Full-text analysis and quality assessment was performed using the National Institute of Health Tool. Data were extracted on study characteristics, prevalence of TIA deficits, and key study findings. Due to significant heterogeneity, meta-analysis was not possible. Twenty-five full-text articles met the review inclusion criteria. There was significant heterogeneity in terms of cognitive tests used, definitions of cognitive impairment and TIA, time points post-event, and analysis methods. The majority of studies used the Mini-Mental State Examination (MMSE) or Montreal Cognitive Assessment (MoCA) (*n* = 23). Prevalence of cognitive impairment ranged from 2% to 100%, depending on the time-point and cognitive domain studied. The MoCA was more sensitive than the MMSE for identifying cognitive deficits. Deficits were common in executive function, attention, and language. No studies assessed diagnostic test accuracy against a reference standard diagnosis of cognitive impairment. Recommendations on cognitive testing after TIA are hampered by significant heterogeneity between studies, as well as a lack of diagnostic test accuracy studies. Future research should focus on harmonising tools, definitions, and time-points, and validating tools specifically for the TIA population.

## 1. Background

Transient ischaemic attacks (TIA) are transient episodes of neurological dysfunction caused by focal ischaemia and hypoxia, where symptoms resolve within 24 h, and there is no evidence of acute infarction (L. Wang et al., 2013). Despite TIA’s transient nature, studies have demonstrated that TIA patients are at greater risk of recurrent stroke and cognitive impairment (L. Wang et al., 2013; Pendlebury & Rothwell, 2019). Cognitive impairment can lead to reduced quality of life (Cumming et al., 2014), and increased dependency, depression, and mortality (Blackburn et al., 2013; Obaid et al., 2020). Cognitive impairment has been identified as a key research priority and unmet need for patients post-stroke (Pollock et al., 2014), and the need to effectively screen and diagnose cognitive dysfunction post-stroke is an increasing priority for clinicians.

The relationship between TIAs and cognitive impairment is not fully understood, and a causal relationship has not yet been established (Van Rooij et al., 2016). It could be that TIAs lead to permanent brain tissue damage, despite the inherent transient nature of symptoms, which could affect cognitive processing. Alternatively, cognitive impairment may have been present prior to the TIA, and unmasked by the event. Vascular risk factors triggering the TIA may also trigger cognitive impairment or, patients post-TIA may also be suffering from delirium, anxiety or depression which manifest as cognitive deficits. Although there is still much to learn, it is important to screen for cognitive impairment following TIA, as well as in stroke patients. Post-stroke cognitive impairment is highlighted as a key priority for assessment in several national and international guidelines (Quinn et al., 2018, 2021), and a similar emphasis should be placed on those post-TIA, given the significant overlap in risk factors and implications for patients.

Cognitive impairment can affect many different neurological functions and processes including executive function, attention, planning, memory, language, and visuospatial function (Swartz et al., 2016). In particular, executive function is commonly affected in vascular-type cognitive dysfunction (e.g. stroke, TIA). Executive function is critical for the execution of many everyday tasks and activities, and therefore the impact of dysexecutive syndromes can be profound. A recent study found almost 40% of TIA patients were unable to drive 6 weeks post-event, due to a combination of executive dysfunction and loss of confidence (Bell et al., 2017). In addition to long—term cognitive impairment, transient dysfunctions in cognition have also been demonstrated post-TIA; up to 40% have reduced cognitive function at 7 days post-event, compared to subsequent testing at one month (Pendlebury et al., 2011).

National and international guidelines recommend cognitive assessment following stroke as a key component of comprehensive stroke care (Quinn et al., 2018; R. Lees et al., 2014). In a systematic review and meta-analysis by Lees et al, no one cognitive screening test was clearly superior in sensitivity and specificity for assessing cognition post-stroke. However, this review did not distinguish between TIA and stroke. Cognitive deficits in TIA may be more subtle than post-stroke, requiring greater sensitivity, particularly for executive dysfunction.

Tools which have been commonly studied to assess cognition post-TIA include the Mini-Mental State examination (MMSE), Montreal Cognitive Assessment (MoCA) and various other neuropsychological test batteries. However, in recent years, cost implications have arisen through copyright and manual and training fees, which may limit the ongoing use of these tests (Burton & Tyson, 2015; Tong et al., 2017). Furthermore, older tests such as the MMSE are further limited by a lack of sensitivity for milder impairments, and a notable absence of testing for executive deficits, making them less applicable to vascular cognitive disorders (Mancuso et al., 2018, 2018; Moorhouse et al., 2010, 2010). The MMSE has been shown to have reduced sensitivity in stroke patients (Nys et al., 2005) and is therefore likely to perform poorly in TIA patients. The MMSE takes~10 min to complete, and a threshold of < 27 is commonly used for the detection of cognitive impairment (Quinn et al., 2018). However, the test properties are affected by age, ethnicity and education, and adjusted thresholds may be required for these groups (Nilsson, 2007). The MoCA has been more widely adopted for vascular cognitive impairment owing to its improved sensitivity for milder deficits and tests of executive function. The MoCA also takes~10 min to complete and a threshold of < 26 is commonly used for detecting cognitive impairment (Cumming et al., 2013; Dong et al., 2010; Godefroy et al., 2011). The MoCA has been demonstrated to be more sensitive than the MMSE at identifying cognitive decline post-stroke (Salvadori et al., 2013), but may still miss subtle impairments. Similarly, MoCA scores will vary between different populations, for example a mean of 23.4 was found by Rossetti et al., which is lower than the original cut-off of 26; it was thought that this was because Rosetti et al sampled a wider population from multiple ethnic backgrounds, compared to the original study which only tested the instrument on healthy Canadian controls (Nasreddine et al., 2005; Rossetti et al., 2011).

Both the MMSE and MoCA thresholds can be problematic if clinicians base a diagnosis of cognitive impairment solely on an assessment test score because education, previous history, age, sex, ethnicity and other factors should also be taken into account.

Although widely studied, neuropsychological test batteries are less feasible in acute settings, due to the length of administration and requirement for specialist training.

The last review of cognitive impairment and assessment specifically in a TIA population was conducted in 2016 (Van Rooij et al., 2016), although numerous reviews have been published in stroke populations since then (Mijajlovic et al., 2017; Quinn et al., 2018, 2021; R. A. Lees et al., 2017). Given the unique challenges to cognitive assessment in TIA populations (e.g. milder deficits, limited time in the TIA clinic), this patient group warrants a review in its own right, as the test properties may need adaptation. Therefore, the aim of this systematic review is to provide a comprehensive and up-to-date review of the prevalence of cognitive impairment post-TIA, the evidence for cognitive assessment tools in a TIA population, and to assess which tools should be recommended for use in clinical practice.

## 2. Methods

### 2.1. Searches

**Figure 1.**
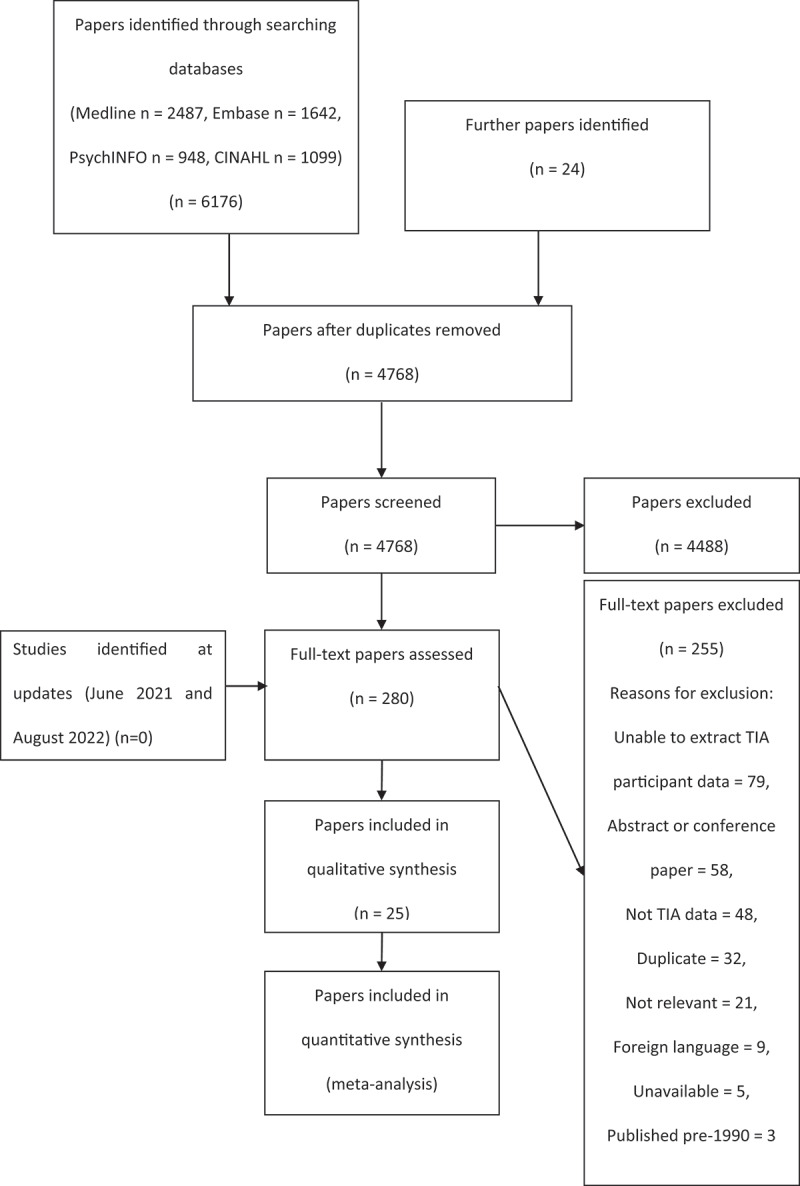
PRISMA flow diagram of articles used in this review with reasons for exclusion.

This review was conducted in accordance with the PRISMA guidance for reporting systematic reviews. The full review protocol was registered on PROSPERO (CRD42019131229). Medline, Embase, PsychINFO and CINAHL databases were searched from inception to June 2021 using the strategy in supplementary information (S1). Reference lists of the included articles were screened for additional relevant material. Only full-text, English language, and adult human studies where TIA patient data could be successfully extracted were considered. Included studies had to use a cognitive assessment recorded after a TIA, however, were not limited to a specific cognitive test and included neuropsychological test batteries. Articles were excluded if they were abstracts, conference papers, posters, single case series or a mixed population of TIA and stroke patients, where TIA specific data could not be extracted. Figure 1 shows a PRISMA flow diagram for the papers identified through the database searches. The search was completed in July 2019 and updated again in both June 2021 and August 2022.

### 2.2. Data extraction and analysis

All papers were screened by title and abstract against the inclusion and exclusion criteria by a single reviewer (AH) and a random subsection (10%) were independently screened by a second reviewer (LB). Additional papers generated from the updated search were independently reviewed by two reviewers (AH and TC). All full-text papers were then independently evaluated by two reviewers (AH, LB). Any disagreements were resolved by discussion and final decisions regarding eligibility were made after a review of the full text. Data extraction was performed by a single reviewer (AH) in Microsoft Word for Windows. None of the included articles met the pre-defined criteria for meta-analysis which were: at least two studies with sufficient homogeneity in sample population, assessment tool, and timescale. Studies were considered eligible for meta-analysis where at least two studies examining the same cognitive test, with the same outcome measures (e.g. mean and standard deviation), at the same time point post-event, and in a comparable population (i.e. same definition of TIA). We also considered the use of standardised mean differences to combine test score from different tools, however there were insufficient studies conducted at the same time point with a comparable definition of TIA for this to be achieved. Furthermore, test scores were often reported for sub-domain tests contained within a broader neuropsychological test battery and these were not considered eligible for meta-analysis with global scores obtained from screening tools (e.g. MoCA, MMSE), given the test properties of an in-depth battery or single sub-domain score are likely to reflect different aspects than global cognitive function from a brief screening test. Therefore, a narrative synthesis was conducted for all included studies.

### 2.3. Risk of bias (quality assessment)

Risk of bias was assessed using the National Institutes of Health (NIH) tool for observational cohort and cross-sectional studies. The quality assessments were made independently by two reviewers (AH, LB, or TC), with excellent agreement between reviewers (k = 0.78, *p* < 0.05). Any disagreements were resolved through discussion. Risk of bias tables were made using the domains NIH tool and are presented in Table 2.

## 3. Results

### 3.1. Summary of included studies

A narrative synthesis of the included studies is presented in this section, grouped by time between TIA and cognitive assessment. Studies were divided into acute (0 to 14 days), post- acute (14 days to 6 months), or chronic (after 6 months) timings of cognitive assessment. Meta-analysis was not possible due to the significant heterogeneity in methodology and outcome measures, as well as inadequate presentation of numerical data. This heterogeneity was particularly evident in varying timeframes between TIA and assessment. Twenty-five studies met the inclusion criteria, and the key characteristics of included studies are summarised in Table 1. Most of the studies used the Mini-Mental State Examination (MMSE) or Montreal Cognitive Assessment (MoCA) (n = 24). The MoCA was more sensitive than the MMSE for cognitive deficits. Deficits were found to be more common in executive function, attention, and language. Prevalence of cognitive impairment ranged from 2–100%, mean = 40.3, standard deviation = 26.3.

**Table 1.** Study characteristics for included manuscripts

Study	Prevalence of cognitive impairment	Setting	No. TIA	Setting	Cognitive assessment tool	Time of cognitive testing post TIA	Control group	Main conclusion
**Unspecified**								
Gao et al. (2014)	NR	China	13	Inpatient and outpatient	MMSE, NUCOG, (modified Chinese versions)	NR	260 healthy controls 44 dementia 144 epilepsy	MMSE scores did not differ between TIA and stroke (25 vs 26), whereas NUCOG scores were lower in stroke than TIA (77 vs 83, *p*=0.004).
Guo et al. (2014)	NR	China	35 TIA and ipsilateral carotid stenosis	Unclear	MoCA, modified Chinese version	NR	35 healthy controls	TIA patients had a significantly lower MoCA scores than the control group (24 vs 26, *P*= 0.001).
Guyomard et al. (2011)	57%	UK	68	Inpatient and outpatient	MoCA	NR	68 controls without vascular disease or risk factors	MoCA scores were significantly lower in TIA group compared to non-TIA (25 vs 28, *p*<0.0001).
Hoffmann et al. (2009)	36%	USA	309	Unclear	COCONUTS	NR	1796 stroke	Cognitive impairment was present in 87% of stroke vs 36% of TIA.
Rao et al. (1999)	40%	UK	25	Inpatient and outpatient	CAMCOG, MMSE, NPTB	NR	25 PVD 25 stroke 25 controls	TIA cohort had global impairment on MMSE (*p*=0.003) and frontal lobe dysfunction (*p*=0.002). Frontal lobe impairment was an independent predictor of global cognitive impairment in TIA.
Swartz et al. (2016)	9%	Canada	132	Outpatient	MoCA, NPTB	NR	133 stroke 135 other vascular or non-vascular	MoCA scores did not differ between TIA, stroke, and other conditions (25 vs 25 vs 26).
Zinn et al. (2007)	56–100%	USA	9	Inpatient	NPTB	NR	47 stroke patients 10 patients with risk factors	Rates of impairment on neuropsychological tests were ~50% in stroke and TIA. Tests assessing working memory, cognitive flexibility, and processing speed were more impaired in stroke and TIA patients compared to patients with risk factors only.
**Acute - Within 7 days**								
Shopin et al. (2013)	NR	Israel	138 TIA 316 stroke patients	Inpatient	MoCA, MindStream	Within 3 days of event	None	MoCA and MindStream scores were lower in stroke than TIA (23 vs 24, *p*=0.001). MoCA and MindStream were correlated (*r*=0.6, *p* < 0.001). MoCA identified more subjects with low scores (<26) compared to MindStream (70.6 vs. 15.7%).
Sörös et al. (2015)	5–40%	Canada	140	Outpatient	MMSE, NPTB	Within 1 week of TIA	None	57% of patients were impaired on one or more tests. ~30% were impaired on two or more tests. Cognitive impairment was identified most frequently on tests of executive function (TMT-A and B 31 and 40% respectively). Only 5% of patients were impaired on the MMSE.
Su et al. (2018)	NR	China	25	Outpatient	MoCA	1 week post-event	25 healthy controls	MoCA scores did not differ between TIA patients and controls (26 *vs* 27, *P*=0.054).
L. Wang et al. (2013)	21–98%	China	97	Inpatient	MoCA (Chinese version)	Within 7 days	100 healthy controls	Impaired verbal fluency (97.93%), memory recall (91.75%), abstraction (84.53%), and visuospatial/executive abilities (79.38%). To a lesser degree, TIA patients also showed impairment in attention (50.52%), naming (20.62%), and orientation (20.62%).
**Acute - Within 14 days**								
Blackburn et al. (2013)	23–46%	UK	13 TIA and 37 stroke	Inpatient	MoCA, MMSE	Within the last 14 days	None	46% of TIA were impaired on the MoCA vs 23% on the MMSE. MoCA and MMSE scores were lower in stroke than TIA (20 vs 24, and 26 vs 28 respectively).
**Post-acute - Within 1 month**								
J. Wang et al. (2016)	50%	China	50 (25 with VCI, 25 no-VIC or control)	Inpatient	MoCA, MMSE	4 weeks post-TIA	25 healthy controls	CTP changes greater in patients with cognitive impairment at 4 weeks post-TIA. Negative correlation between MoCA score and CTP parameters in patients.
**Post acute - Within 3 months**								
Van Rooij et al. (2014)	38%	NL	107	Inpatient and outpatient	NPTB	Within 3 months of event	81 healthy controls	TIA patients performed worse than controls on all domains except episodic memory. EF: OR 3.5 (95%CI: 0.7–16.7), WM: OR 22.5 (95%CI: 2.9–174.3). Impairment of ≥1 cognitive domain was present in 38% of patients with TIA, OR: 5.9 (95%CI: 2.4–14.5).
**Chronic - Within 6 months**								
Bakker, Klijn, Jennekens-Schinkel, Van Der Tweel, Tulleken, et al. (2003)	54%	NL	39 TIA with carotid artery stenosis	Outpatient	NPTB	Within 6 months	46 spouses/siblings	54% of TIA vs 8% of controls were classified as cognitively impaired.
Pendlebury, Mariz, et al. (2012)	83%	UK	142	Outpatient	MoCA, MMSE	At least 6 months post-event	207 stroke 107 memory clinic referrals	Mean cognitive scores were lower in stroke compared to TIA patients (MMSE: 28 vs 28, MoCA: 23 vs 25). No difference between TIA only patients and memory clinic referrals (MMSE: 29, MoCA: 26).
**Multiple time points**								
Bakker et al. (2004)	39%	NL	26 retinal or cerebral TIA patients	Outpatient	Neuropsychiatric battery: general intelligence	Baseline, 6 months and 12 months	73 healthy controls 47 stroke	39% of patients with TIA were cognitively impaired at baseline. In the total patient group (stroke and TIA), only patients without a further TIA during follow up period improved in cognitive functioning at 6 and 12 months follow up. In patients with a TIA in follow up period, there was no significant difference between baseline and follow up.
Bivard et al. (2018)	NR	Australia	50	ED or rapid referral TIA service	MoCA	At presentation, 90 days	None	Patients with an anterior perfusion lesion on acute imaging had a significant decrease in MoCA score between baseline and day 90 (*p*=0.027), which may be related to the volume of thalamic atrophy (*R*^2^=0.28; *P*=0.009).
Charoenkitkarn et al. (2009)	NR	Thailand	52	Outpatient and ED	NPTP	3, 10 and 30 days post TIA	52 controls who had experienced minor surgery	TIA patients had worse cognition in all three domains, at all time points compared to controls, which continued after 30 days. Performance was worst at day 10, improving at day 30.
Kjörk et al. (2016)	30–41%	Sweden	44, then 23 at follow up.	Outpatient	MoCA	1 month and 9 months post event	None	Cognitive impairment was present in 40% (*n* = 44) after 1 month and 30% (*n* = 23) after 9 months Cognitive impairment was present in 40% (*n* = 44) after 1 month and 30% (*n* = 23) after 9 months Cognitive impairment was present in 40% of TIA after 1 month, and 30% after 9 months.
Pendlebury et al. (2013)	29%	UK	42	Outpatient	MoCA, MMSE, NPTP	1 or 5 years follow-up	49 stroke	MCI was more likely in stroke than TIA (55% vs 29%, OR =3.06, 95% CI= 1.28–7.36, *p* = 0.01). MCI-modified threshold: 24% vs 12%, OR = 2.40, (95% CI = 0.77–7.50, *p* = 0.13) Single-domain was more likely than multi-domain MCI in TIA patients.
Pendlebury, Markwick, et al. (2012)	2–21%	UK	44	Outpatient	MoCA, MMSE, ACE-R, NPTP	1 year or 5 year follow up	55 stroke	Between 2–21% of TIA participants were impaired on various subsets of a NPTB compared to 2–20% of stroke patients.
Pendlebury et al. (2011)	19%	UK	206 mixed TIA and stroke patients	Outpatient	MMSE	1–7 days, >7 days post-event and at 1 month	47 people without cerebrovascular disease	The rate of cognitive impairment was higher in TIA and stroke patients seen acutely than in those seen after 7 days (39% vs 19%, OR, 2.72, 95% CI: 1.43–5.19; *P*=0.002), and in cerebral TIA and stroke patients seen acutely versus non-cerebrovascular patients (39% vs 21%, *P*=0.004).
Van Rooij et al. (2017)	NR	NL	73	Outpatient	NPTB	Within 7 days, and 6 months	48 transient neurological attack	Executive function performance decreased (−0.23, *P*=0.01), attention improved (0.11; *P*=0.02), processing speed and episodic memory were unchanged. Executive function was worse in those with DWI lesions (−0.26 vs 0.08, *P*=0.048). Cognitive function was the same in TIA and TNA patients over time.
Volonghi et al. (2013)	8–63%	UK	181	Outpatient	MoCA, MMSE, TICSm	1 and 5 years post TIA	216 ACS 218 stroke	ACS patients had poorer cognitive function than TIA patients at 1 year (MMSE: 27 vs 28, *p*<0.0001; OR=2.14, 95%CI 1.11–4.13). Memory and language versus frontal/executive subtests were relatively more impaired in ACS than TIA and stroke.

**Abbreviations**: ACE-R = Addenbrooks cognitive examination, ACS= acute coronary syndrome, CAMCOG = Cambridge Cognition examination, COCONUTS= comprehensive cognitive neurological test in stroke, CTP= CT perfusion, CVD= cerebrovascular disease, EF= executive function, MCI = Mild cognitive impairment, MCST = modified card sorting test, MMSE = Mini-mental state examination, MoCA = Montreal Cognitive Assessment, MRI = Magnetic Resonance Imaging, mTICS = modified Telephone interview of cognitive status, NL= Netherlands, NPTB= neuropsychological test battery, NR = Not reported NUCOG = Neuropsychiatry unit cognitive assessment tool, OR= odds ratio, OXVASC = Oxford Vascular Study, PVD= peripheral vascular disease, SD = standard deviation, TMT = trail making test, VCI = UK= United Kingdom, USA= United states of America, Vascular Cognitive Impairment, WM= working memory. Pendlebury, Mariz, et al. (2012) (Pendlebury, Mariz, et al., 2012), Pendlebury, Markwick, et al. (2012) (Pendlebury, Markwick, et al., 2012).

#### 3.1.1. Cognitive outcomes

Of the included studies: 14 used the MoCA; ten the MMSE; eleven a neuropsychological battery; and seven used other cognitive assessment tools, these were: Mindstream, Cambridge Cognition examination (CAMCOG), Comprehensive Cognitive Neurological Test in Stroke (COCONUTS), Neuropsychiatry unit cognitive assessment tool (NUCOG), modified Telephone interview of cognitive status (TICS-m), ACE-R, Cognitive Failures Questionnaire (CFQ). Studies defined cognitive impairment variably: six studies used a cut-off based on published norms or a set number of standard deviations below the mean, five did not report a definition, nine used a specified threshold on a cognitive test, two used diagnostic criteria, one used a consensus based on imaging, scores, and history, and the remainder used other methods. Furthermore, different studies took different potential confounders for cognitive impairment into account. 15 studies adjusted their findings for years of education, 12 studies adjusted for age, 7 adjusted for sex and only 4 adjusted for previous cognitive ability.

#### 3.1.2. Participant populations

Thirteen studies included both TIA and stroke patients (presenting data separately), whereas twelve studies recruited TIA patients only. Five studies had no control group, and the remaining studies had control groups comprised of: healthy individuals; those with dementia or epilepsy, peripheral vascular disease, vascular risk factors, transient neurological attack, other vascular or non-vascular disease, acute coronary syndrome or patients referred to memory service. The definition of TIA was variable with twelve studies providing no clear definition, eleven providing a time-based definition based on physical symptoms improving within 24 h, and the remainder a tissue-based definition i.e., the TIA caused transient brain ischaemia but no infarction. Most of the studies did not report how the TIA diagnosis was made, except that patients were seen in a specialist clinic or diagnoses were made according to WHO criteria. Depression, anxiety or stress are all important factors to consider in TIA. They can all occur secondary to a TIA and can all adversely affect cognitive assessment scores (Müller et al., 2021). Nine of the studies specifically mentioned at least one of these in their exclusion criteria. Five studies excluded patients who were known to have depression (Charoenkitkarn et al., 2009; Gao et al., 2014; Guyomard et al., 2011; L. Wang et al., 2013; Pendlebury et al., 2011). Two studies mentioned more broadly excluding conditions which could lead to cognitive disturbance (Bivard et al., 2018; Blackburn et al., 2013). The rest did not mention them at all.

#### 3.1.3. Settings

Subject recruitment differed between studies. Twelve studies recruited patients from outpatient settings, five from inpatient settings, four from either in- or outpatient settings, two from emergency departments or rapid referral clinics, and two were unclear. The time interval between cognitive testing and event varied considerably between studies; the majority measured cognitive skills at presentation or within one week (*n* = 5). However, others tested within 14 days, within 30 days, at or within three months, within six months, or at multiple time points. Furthermore, eight studies did not specify a time, or it was unclear. Only two studies reported the accuracy of the cognitive tests in terms of sensitivity and specificity for MCI diagnosis, but none for a clinical dementia diagnosis.

### 3.2. Quality assessment

The median quality assessment score was 7 (inter-quartile range: 7–8). Only seven studies reported the number of people meeting eligibility criteria for study inclusion, and 12 recruited all participants from the same population over the same time period with uniform inclusion and exclusion criteria for all participants. Only two studies reported a sample size justification, and only three measured the exposure status (repeated TIAs) over time. Eight studies reported a loss to follow-up of less than 20% of the baseline sample, and the remainder either did not report drop-out rates or had>20% loss of participants over the study. Seven of the studies adjusted for differences in baseline demographics or key confounding variables (e.g., age, education level). Studies which had an unspecified time point for measurements had a mean score of 6.14 compared to 7.94 in those where the time point was specified. Table 2 summarises the quality assessment for included studies, and the full checklist can be seen in Appendix 1.

**Table 2.** Quality assessment of included studies using the National Institutes of Health scale for observational cohort and cross-sectional studies

Study	1	2	3	4	5	6	7	8	9	10	11	12	13	14	Total
Bakker et al. (2004)															11
Bakker 2003															8
Bivard et al. (2018)															7
Blackburn et al. (2013)															6
Charoenkitkarn et al. (2009)															9
Gao et al. (2014)															7
Guo et al. (2014)															5
Guyomard et al. (2011)															7
Hoffmann et al. (2009)															5
Kjörk et al. (2016)															9
Pendlebury et al. (2011)															8
Pendlebury, Mariz, et al. (2012)															7
Pendlebury, Markwick, et al. (2012)															8
Pendlebury et al. (2013)															11
Rao et al. (1999)															7
Shopin et al. (2013)															7
Sörös et al. (2015)															7
Su et al. (2018)															5
Swartz et al. (2016)															6
Van Rooij et al. (2017)															11
Van Rooij et al. (2014)															7
Volonghi et al. (2013)															8
J. Wang et al. (2016)															7
L. Wang et al. (2013)															7
Zinn et al. (2007)															6

Grey= present, white= absent. Full checklist can be seen in Appendix 1. Pendlebury, Mariz, et al. (2012) (Pendlebury, Mariz, et al., 2012), Pendlebury, Markwick, et al. (2012) (Pendlebury, Markwick, et al., 2012).

### 3.3. Acute measurements

In this review, acute measurements were taken to be those that occurred within the first fourteen days of presentation as a TIA. Nine studies tested cognition at or within one week of presentation. Of these nine studies, three used the MoCA, three used a neuropsychological battery alone, and one used the MMSE. One used the MMSE alongside a test battery, and one used the MoCA and Mindstream. Seven studies out of the nine found cognitive impairment in their TIA patient cohort based on a pre-determined cut-off score on a cognitive test, despite using varied cognitive tests as described above. The prevalence of impairments varied between 30% (impairments in two or more domains (Sörös et al., 2015)) and 97% (verbal fluency (L. Wang et al., 2013)), depending on the cognitive domain or test. Wang et al found impairments particularly in the following MoCA subdomains: verbal fluency (97%), memory recall (92%), abstraction (85%) and visuospatial abilities (80%), and to a lesser degree attention (50%), naming (21%) and orientation (21%) (L. Wang et al., 2013). Similarly, Charoenkitkarn et al found poorer test scores in three domains of a neuropsychological test battery compared to controls at presentation: attention, working memory, and learning and memory (Charoenkitkarn et al., 2009). Compared to Mindstream, the MoCA (threshold<26) was found to be more sensitive for cognitive deficits post-TIA, identifying 70.6% as impaired compared to 15.7% (Sörös et al., 2015). However, one study found no significant difference in MoCA scores between TIA patients (25) and healthy controls (25) (*p* = 0.054) (Su et al., 2018). Unlike the others this study excluded all patients over 65 and those with a history of cognitive impairment which may have accounted for their findings. In a study by van Rooij et al, all executive function was worse in patients with a diffusion weighted lesions on MRI, compared to those without (Van Rooij et al., 2017). These diffusion weighted lesions were defined as new, less than 5 mm and seen 7 days post-TIA. Compared to the TIA cohort with no measurable lesions this group had significantly lower scores in the executive function section of the MMSE. However, this was not seen after 6 months.

A further three studies performed cognitive testing within 14 days of TIA and all three studies reported cognitive impairment, although to differing degrees (19–46%). Blackburn et al reported 46% and 23% of TIA participants were impaired on the MoCA and MMSE, respectively (Blackburn et al., 2013). Pendlebury et al found that 19% of TIA and stroke patients were impaired on the MMSE when testing more than 7 days after TIA, although this was reduced from 39% at presentation (Pendlebury et al., 2011). Cognitive impairment was also more common amongst patients with a cerebrovascular versus non-cerebrovascular diagnosis (Pendlebury et al., 2011). When comparing studies there is generally a decrease in the degree of cognitive impairment overtime. However, Charoenkitkarn et al examined TIA 10 days post-event and found cognition was worst at day ten relative to day 3 and 30 in three cognitive domains (attention, working memory, and learning and memory) compared to controls (Charoenkitkarn et al., 2009). This was a study of 52 individuals from primary and tertiary care, they replaced those lost to follow up after 10 days due to a second neurological event with four more individuals.

### 3.4. Post-acute and chronic measurements

Post-acute and chronic measurements were any studies which measured cognition more than two weeks or 6 months after the TIA. Studies were divided at these stages as previous reviews have also divided studies at these points, and to help distinguish between transient changes that occur in the first 14 days compared to those which persist afterwards (Van Rooij et al., 2016). 6 months was chosen as the chronic cut-off as this is typically a measure of chronic stroke (Liao et al., 2018). These studies were heterogeneous in their timings and the types of tests that they used made direct comparisons difficult. Four studies performed cognitive testing one-month post-TIA. Of these four studies, one used a neuropsychological battery compared to minor surgery patients (Charoenkitkarn et al., 2009), one used the MoCA with no comparison group (Kjörk et al., 2016), one used the MMSE compared to non-cerebrovascular patients (Pendlebury et al., 2011), and one used the MoCA and MMSE compared to healthy controls (J. Wang et al., 2016). All four studies identified cognitive impairment in TIA patients (19–50%). Kjork et al did not have a comparison group, but identified that cognitive impairment was present in 40% of patients post-TIA at 1 month (Kjörk et al., 2016). Charoenkitkarn et al demonstrated function in three cognitive domains (attention, working memory and learning and memory) was poorer in TIA patients compared to the minor surgery patients (Charoenkitkarn et al., 2009).

Two further studies performed cognitive testing at or within three months of presentation. One examined TIA patients at presentation and subsequently at 3 months, finding patients with an anterior perfusion lesion TIA on acute imaging had a significant decrease in MOCA score between baseline and day 90 (*P* = 0.027), which may be related to the volume of thalamic atrophy (*R*^2^ = 0.28; *P* = 0.009) (Bivard et al., 2018). The other was performed by Van Rooij et al and demonstrated worse performance on neuropsychological battery for TIA patients compared to controls in each cognitive domain, except episodic memory (Van Rooij et al., 2014). The highest impairment rates were present in the domains of working memory and attention (odds ratios 3.5–22.5), whereas episodic memory was relatively preserved (Van Rooij et al., 2014). Impairment of ≥ 1 cognitive domain (excluding episodic memory) was present in 38.3% of patients with TIA, with an associated age- and sex-adjusted odds ratio of 5.9 (95% confidence interval, 2.4–14.5) (Van Rooij et al., 2014).

An additional four studies performed cognitive testing within or at six months post-TIA, demonstrating cognitive impairment in 54–82% of patients. Two of these studies used a neuropsychological battery compared to healthy controls and found that TIA patients had higher incidence of cognitive impairment, (54% versus 8%) compared to controls (Bakker et al., 2004; Bakker, Klijn, Jennekens-Schinkel, Van Der Tweel, Tulleken, et al., 2003). In contrast, Pendlebury used the MoCA and MMSE at least 6 months after the event and did not demonstrate significant difference between TIA patients and their control group which was made up of individuals who had been referred to memory service (MMSE: 28.4 vs 28.5, MoCA: 24.9 vs 25.5) (Pendlebury, Markwick, et al., 2012). This could be due to a longer follow-up period (at least six months), or that TIA represents a similar “at risk” population to those referred to a memory service. Two of the studies which tested after 6 months demonstrated that rates of cognitive impairment were greater in stroke compared to TIA (Bakker et al., 2004; Pendlebury, Markwick, et al., 2012). This may have been due to greater deficits or potentially because the test batteries were less sensitive to deficits in TIA. Only one study in this group of four measured cognitive impairment using the MoCA and it found impairment in 30% at nine months post-event (Kjörk et al., 2016).

Four studies followed patients up from between one- and five-years post-event. Bakker et al used a neuropsychological battery in comparison to healthy controls (Bakker et al., 2004). Volonghi et al used the MoCA, MMSE and TICS-m in comparison to stroke patients and acute coronary syndrome (ACS) patients (Volonghi et al., 2013). Pendlebury et al used the MoCA, MMSE, Neuropsychological battery and ACE-R in comparison to stroke patients (Pendlebury et al., 2013; Pendlebury, Mariz, et al., 2012). Volonghi et al tested at both one- and five-years post-event, whereas Pendlebury et al tested between one and five years (mean 3.1 ± 1.9 years) (Pendlebury et al., 2013). All four studies reported cognitive impairment in TIA patients (2–63%).

As reported at earlier time points, Pendlebury et al demonstrated that cognitive impairment was significantly higher in stroke patients compared to TIA patients (55% compared to 29%, OR = 3.06, 95% CI = 1.28–7.36, *p* = 0.01) when tested 12 months post-event (Pendlebury et al., 2013). Volonghi et al also reported worsening cognitive impairment in stroke and ACS patients compared to TIA patients at one year (Volonghi et al., 2013). Interestingly, moderate/severe cognitive outcomes (MMSE<24) were worse at one year in ACS compared to TIA patients (MMSE: 26.6 vs 27.6, *p* < 0.0001; OR = 2.14, 95% CI 1.11 to 4.13), with a similar trend at 5 years (Volonghi et al., 2013). This may have been due to increased risks of stroke post-MI, Jernberg et al. (2015) found that 2.4% of patients in their retrospective cohort study had a stroke within 1 year of a myocardial infarction (Jernberg et al., 2015). Bakker et al. (2004) demonstrated that impairment was worse in TIA patients who had suffered a further TIA in the follow-up period, compared to TIA patients who had not (Bakker et al., 2004).

### 3.5. Varying timepoints

Six studies compared cognition at varying time points. There was no consensus as to whether cognition declined or improved over time. Two of the six studies reported a decline in cognition as time progressed, they had a mean quality assessment score of 8. Bivard et al showed a significant decline in MoCA score between presentation and 90 days (*P* = 0.027), as did Charoenkitkarn et al from presentation to 3, to 10 days (Bivard et al., 2018; Charoenkitkarn et al., 2009).

In contrast, three of the six studies reported an improvement in cognition as time progressed. They had a mean quality assessment score of 9.3. Pendlebury et al reported that baseline MMSE scores significantly improved between seven days and one month, and the rate of cognitive impairment was higher in TIA and stroke patients at presentation compared to those seen after 7 days (39% versus 19%; OR, 2.72; 95% confidence interval 1.43–5.19; *P* = 0.002) (Pendlebury et al., 2011). Kjörk et al showed the incidence of cognitive impairment based on MoCA score cut-off was higher at one month (44%) compared to nine months post event (30%) (Kjörk et al., 2016). Bakker et al demonstrated that in the total patient group (stroke and TIA), there was improvement in cognitive functioning tested via the neuropsychological battery in patients without a further TIA during follow up period at 6 and 12 months follow-up compared to baseline (Bakker et al., 2004). However, in patients with a subsequent TIA in the follow up period, there was no significant difference between baseline and follow up (Bakker et al., 2004). The differences between these studies could be explained by the fact that Bivard et al and Charoenkitkarn et al’s initial assessments were within 1 day or 3 days; compared to the other studies where the first assessment was at 7 days, 1 month and 6 months. This suggests that any decline in cognition is not measurable in the first few days after TIA.

One study of these six varying timepoint papers reported variable changes depending on the cognitive domain: executive function decreased over time, whereas attention improved, and processing speed and episodic memory remained stable (Van Rooij et al., 2017). Changes in cognitive function over time were comparable between TIA patients and those classified more broadly as transient neurological attack (Van Rooij et al., 2017). They tested their patients at some point within the first 7 days of diagnosis and again after 6 months.

### 3.6. Unspecified timepoints

Seven studies did not specify the time at cognitive testing (Gao et al., 2014; Guo et al., 2014; Guyomard et al., 2011; Hoffmann et al., 2009; Rao et al., 1999; Swartz et al., 2016; Zinn et al., 2007). All seven studies demonstrated cognitive impairment in TIA patients (9–100%, median 48%), compared to either healthy controls, peripheral vascular disease, and non-TIA patients. Guyomard et al found cognitive impairment (MoCA<26) was more prevalent amongst TIA patients than controls (57% vs 0%; MoCA scores: 25 ± 1.89 vs 28.4 ± 1.16, *p* < 0.005), with poorer visuospatial, attention, language, memory, conceptual thinking and orientation (Guyomard et al., 2011). In addition, vascular risk factors were more common amongst those with cognitive impairment (current smokers, previous myocardial infarction), and three or more risk factors was associated with cognitive impairment (*p* = 0.03) (Guyomard et al., 2011). Zinn et al found cognitive impairment was prevalent (~50%) in a combined stroke and TIA group (Zinn et al., 2007). Although Swartz et al found 9% of TIA patients had cognitive impairment, there was no significant difference in MoCA scores between stroke, TIA, or other vascular and non-vascular conditions (Swartz et al., 2016). This may have been because they used a lower cut-off score of 22 on the MoCA than other studies.

Three of the seven studies found that the degree of cognitive impairment was worse in stroke patients compared to TIA patients. However, in one of these three this was only in the trail making and verbal fluency tests (Rao et al., 1999). In another, Zinn et al found no significant difference in executive function between stroke and TIA patients (Zinn et al., 2007).

### 3.7. Test comparisons

Eight studies commented on differences between cognitive tests. Four studies found the MoCA was more sensitive for the detection of cognitive impairment when compared to the MMSE. For example, Blackburn et al demonstrated that 70% of patients were impaired on the MoCA versus 30% impaired on the MMSE (Blackburn et al., 2013), and Shopin et al demonstrated that the MoCA was more effective compared to MindStreams at identifying cognitive impairment in TIA patients (70.6 vs. 15.7%) (Shopin et al., 2013).

A further two studies found other cognitive tests superior to the MMSE. For example, Gao et al found that MMSE scores did not differ between neurological diseases (e.g. TIA/Stroke) and healthy controls, whereas NUCOG was able to differentiate between stroke and TIA (Gao et al., 2014). Soros et al identified 57% of patients were impaired on at least one of the neuropsychological battery tests, and nearly one-third impaired on two or more tests, compared to 5% on the MMSE (Sörös et al., 2015). Only one study in the three comparing MoCA to a neuropsychological test battery found the MoCA sensitivity to be lower (60%) (Swartz et al., 2016). This could again, have been due to the lower MoCA cut-off score used.

### 3.8. Test accuracy

Four studies examined the test accuracy of cognitive tools to detect cognitive impairment or dementia, but three of these reported data for a combined TIA-stroke population meaning that individual TIA data could not be ascertained. Pendlebury et al reported test accuracy data for the MoCA using data from the OXVASC cohort (Pendlebury et al., 2013). In TIA patients, the areas under the curve for the detection of MCI was 0.81–0.91 for the MMSE, and 0.81–0.85 for the MoCA, depending on the definition and sub-type of MCI (i.e., single versus multi-domain) (Pendlebury et al., 2013).

### 3.9. Brain imaging

#### 3.9.1. Structural

Six studies examined structural brain imaging in relation to cognitive outcomes in TIA. In one study there was no difference between brain volumes or cognition between TIA and control patients (Su et al., 2018). Similarly, Bakker et al found cognition over 6–12 months was not related to either subcortical or white matter lesions, despite 39% of patients experiencing cognitive impairment at baseline, which was persistent in those with recurrent events over this time period (Bakker et al., 2004). These two studies had a mean of 6.5 on the quality assessment tool. Both studies recruited patients from outpatient clinics.

However, the four other studies demonstrated significant associations between structural imaging changes and cognition in TIA. These studies had a mean of 7.5 on the quality assessment tool. Three of the four recruited patients from inpatient settings. Lesions on diffusion weighted imaging were associated with poorer executive function over six months (Van Rooij et al., 2017), and increased diffusivity and reduced fractional anisotropy negatively correlated with MoCA scores (Guo et al., 2014). Furthermore, thalamic volume change over 90 days was associated with changes in MoCA scores (Bivard et al., 2018). White matter hyperintensities, but not silent brain infarcts or oedema, were associated with poorer executive function within three months of TIA (Van Rooij et al., 2014). These discordant results could be a partial reflection on the source of patient recruitment. Those in an inpatient setting are more likely to have greater severity of symptoms at presentation and therefore, changes on brain imaging.

#### 3.9.2. Functional

Five studies examined functional imaging outcomes in relation to cognitive function after TIA. Su et al found task activation was increased to a working memory task on functional magnetic resonance imaging (MRI) across several brain regions at both one week and three months post-event (Su et al., 2018). However, these changes declined over time, suggesting they represent early compensation, although functional changes were found to persist beyond the recovery in cognitive function (Su et al., 2018). Three of the five studies examined measures of brain perfusion using transcranial Doppler ultrasonography (Bakker, Klijn, Jennekens-Schinkel, Van Der Tweel, Van Der Grond, et al., 2003; L. Wang et al., 2013), and computed tomography perfusion (J. Wang et al., 2016). Peak systolic velocity, reduced CO_2_ reactivity, and increased pulsatility index were found in TIA compared to controls (Bakker, Klijn, Jennekens-Schinkel, Van Der Tweel, Van Der Grond, et al., 2003; L. Wang et al., 2013), but only changes in velocity and pulsatility index were correlated with lower MoCA scores (L. Wang et al., 2013). Two of the five studies demonstrated that the presence of cerebral lactate was associated with poorer cognitive function at 6–12 months (Bakker et al., 2004; Bakker, Klijn, Jennekens-Schinkel, Van Der Tweel, Van Der Grond, et al., 2003). Furthermore, lactate was associated with greater MRI lesions and lower CO_2_ reactivity, but neither of these latter variables were associated with cognition (Bakker, Klijn, Jennekens-Schinkel, Van Der Tweel, Van Der Grond, et al., 2003). Finally, Wang et al demonstrated reduced cerebral blood flow with computed tomography perfusion, as well as peak and transit times, with only the latter being negatively correlated with MoCA scores. (J. Wang et al., 2016). Reduced cerebral blood flow or velocity was seen with both transcranial Doppler ultrasound and computed tomography perfusion.

## 4. Discussion

### 4.1. Summary of results

In summary, most studies included in this review demonstrated cognitive impairment in TIA populations. The prevalence estimates varied from 2% to 100%, depending on the setting, tools used, and definition of cognitive impairment. Most studies in the review used the MMSE or MoCA to assess cognition, with fewer studies investigating neuropsychological test batteries. Studies in the review were case-control or cohort design and no studies in the review formally tested the accuracy of cognitive assessment tools in a TIA population. Studies used different thresholds to diagnose cognitive impairment which could significantly affect the prevalence estimates reported.

### 4.2. Prevalence and trajectory of cognitive impairment

Cognitive impairment prevalence estimates varied significantly between studies included in this review and compared to the review conducted by van Rooij in 2016 they were wider ranging (Van Rooij et al., 2016). This is likely due to heterogeneity in respect of timing of assessments, the tools used to identify cognitive impairment, and the test thresholds and definitions for cognitive impairment used in the individual studies. Rates of impairment were higher with the MoCA and neuropsychological test batteries when compared to the MMSE (Blackburn et al., 2013; Gao et al., 2014; Shopin et al., 2013; Sörös et al., 2015). This is in keeping with a previous review on this topic identifying higher reported rates with the MoCA and TICS-m (Van Rooij et al., 2016), and is likely to be due to the inclusion of different cognitive domains, in particular those that are affected in vascular-type impairments (Dichgans & Leys, 2017; Quinn et al., 2021). Executive function is a key cognitive domain that is impaired in vascular cognitive impairment (Dichgans & Leys, 2017; Povroznik et al., 2018), and in milder impairments may be the sole presenting deficit (Moorhouse et al., 2010; Pendlebury et al., 2013). As a result, tests such as the MMSE which do not assess these domains are less beneficial in this setting (Dichgans & Leys, 2017), although still considered for use in recent guidelines (Quinn et al., 2021). However, rates of impairment also varied even when the same test was applied and is likely to be due to differences in test application, particularly the threshold used. As expected, cognitive deficits were generally found to be lesser amongst TIA patients than those with stroke, likely due to the more localised and transient ischaemia occurring in the brain. However, longitudinal studies included in this review show that deficits can persist up to 5 years (Volonghi et al., 2013), and patients with early deficits go on to have a higher risk of future cognitive decline (Pendlebury & Rothwell, 2019). Whether these prolonged deficits were solely secondary to TIAs is unclear. In a recent study by Pendlebury et al, the risk of dementia at 1 year post-TIA was 3.5-fold, with an incidence of 5%, and bringing the onset of dementia forward by 2 years (Pendlebury & Rothwell, 2019). Thus, early and transient impairment in cognitive function after TIA should not be viewed as a benign condition. In keeping with this, deficit levels were similar between TIA patients and those referred to a general memory clinic (Pendlebury, Markwick, et al., 2012), suggesting this is a similar at-risk group for undiagnosed dementia. However, recent guidelines produced by the European Stroke Organisation and European Academy of Neurology suggested that targeted rather than widespread screening should be adopted in the post-stroke setting due to lack of available evidence to support this (Quinn et al., 2021).

Impairments were reported by all studies in this review at time-points ranging from early (within 7 and 14 days), to medium (1,3,6, and 9 months), and into the longer term (1 and 5 years). Deficits were most prominent in the early phase, recovering in the medium term, albeit still apparent, and deteriorating in the longer term. The presence of longer-term deficits reflects that patients with TIA represent a high-risk group for future development of dementia (Pendlebury & Rothwell, 2019; Volonghi et al., 2013). This could be as a result of accumulating vascular risk burden, but also the interplay between vascular and amyloid pathology, increasing the risk of Alzheimer’s as well as vascular-type cognitive impairment (Kisler et al., 2017; Nelson et al., 2016; Zlokovic, 2011). Furthermore, in this interval, patients may sustain further cerebrovascular events, and evidence supports worsened cognitive function with cumulative TIAs (Bakker et al., 2004). Studies which specifically assessed the temporal changes in cognition since the event were variable, with some showing worsened and some improved cognition. This is likely to be due to population differences (e.g., higher vascular burden, number of recurrent events), or the time points studied.

### 4.3. Cognitive assessment tools

The majority of studies in this review examined brief, bedside screening tests such as the MMSE and MoCA. Three studies examined translated and adapted versions (Gao et al., 2014; Guo et al., 2014; L. Wang et al., 2013), and one compared to an online cognitive assessment tool (Shopin et al., 2013). In addition to brief screening tests, some, albeit fewer, studies investigated more extensive neuropsychological test batteries. TIA clinics have been widely adopted due to the significant (~80%) reduction in stroke risk afforded by the timely management of key risk factors (Rothwell et al., 2007). The TIA clinic uses a “one-stop-shop” model whereby all clinical assessments, biochemical, and radiological tests are conducted on the same day (Rothwell et al., 2007). Therefore, time afforded to cognitive assessment in this setting is limited, and extensive neuropsychological testing requiring input from expert psychologists is unlikely to be feasible. In a previous review, test batteries were more likely to be conducted with younger patients, and may be less feasible in older, frailer patients who may become fatigued during assessment which can affect cognitive performance (Van Rooij et al., 2016). This is important when considering the increased risk of TIA with increasing age (Giles & Rothwell, 2008), and the association of frailty with both cerebrovascular disease (Palmer et al., 2019; Taylor-Rowan et al., 2019), and increasing age (Buchman et al., 2009).

However, in view of the increasing costs for training, manuals, and licensing associated with the two most common tools in this review (MoCA and MMSE), their widespread application is becoming less viable (Tong et al., 2017). Freely available alternatives (Oxford Cognitive Screen, ACE-III, Free-cog) have not yet been studied in this population. In view of the high prevalence of deficits post-TIA, targeted cognitive screening is recommended in both national and international guidance (Quinn et al., 2018, 2021). However, formal test accuracy studies that examine the sensitivity and specificity of assessment tools relative to a clinical reference standard diagnosis of dementia, are lacking. This results in test thresholds which have been developed and validated for stroke or dementia settings being used in a TIA population, which may not be appropriate. There is increasing interest in the development of computerised cognitive assessment tools such as Mindstream and OCS-Plus, which overcome inconsistencies in test administration and scoring, and are less reliant on culture and language for assessment (Demeyere et al., 2020; Humphreys et al., 2017). However, few studies have assessed computerised-based assessments in the TIA clinic, which may be due to barriers with integrating technology into clinical services.

### 4.4. Cognitive domains affected and neuroimaging markers

Most studies in this review investigated global cognitive impairments as measured by brief screening tools which are not validated to provide detailed testing on cognitive sub-domains. However, some studies used more extensive neuropsychological testing for this purpose. Deficits were commonly reported amongst executive function, visuospatial, verbal fluency, working memory, language, and memory domains, which are similar to those identified in a previous review (Van Rooij et al., 2016). When compared to stroke, deficits tended to be fewer or single rather than multiple domain (Pendlebury et al., 2013), although frontal lobe dysfunction was prominent (Rao et al., 1999; Su et al., 2018; Van Rooij et al., 2014, 2017). This highlights the need for a broad assessment tool to increase the capture of single domain deficits which are likely to be more prevalent in a TIA population, as shown in MRI studies of TIA patients with mild cognitive impairment (Mariani et al., 2007). However, this breadth needs to be simultaneously balanced with the time constraints outlined above. targeting the domains highlighted in this review may provide this balance.

Fewer studies examined neuroimaging markers of cognitive dysfunction post-TIA. Structural findings were inconsistent between studies, particularly regarding the association with white matter hyperintensities and brain volumes. Diffusion weighted imaging may be a better reflection of structural changes occurring post-TIA, and abnormalities using this modality appear to be linked to poorer cognitive outcomes (Guo et al., 2014; Van Rooij et al., 2017). Of the functional imaging markers, cerebral blood flow velocity, pulsatility index, cerebral lactate and transit time were associated with cognitive function (Bakker et al., 2004; Bakker, Klijn, Jennekens-Schinkel, Van Der Tweel, Van Der Grond, et al., 2003; J. Wang et al., 2016; L. Wang et al., 2013). Hyperactivation to a working memory task in one study indicated that functional compensation may occur early after TIA, although these changes resolve more slowly than those in cognitive function (Su et al., 2018).

### 4.5. Limitations and future directions

Since the last review of this topic in 2016, there has been limited improvement in the quality of the literature, despite this review identifying more than twice the number of studies in this area. This highlights the need for better quality research, particularly in respect of the feasibility and validation of cognitive tests for patients presenting to the TIA clinic. This review was limited by significant heterogeneity in the included studies in terms of TIA and cognitive impairment definitions, tools and thresholds used for assessment, time-points investigated, and selection criteria. In fact, despite timing being crucial to the assessment of cognitive function post-vascular events, four studies in this review did not specify a time point at which assessments were conducted. There remains considerable heterogeneity in the case definition of TIAs, which cognitive assessment tools are used, when they should be applied, how often they should be repeated, and who is most at risk. There is still a lack of consensus as to whether TIAs lead directly to cognitive impairment and if so, what is the underlying mechanism. False-positive rates could be mistaken for increased sensitivity in studies, as baseline cognition rates are usually unrecorded. A consensus experimental approach needs to be reached for future research to progress our understanding. Furthermore, only a few of the studies commented on whether patients had concomitant signs of depression or stress during the testing, which could have affected their cognitive assessment scores.

## 5. Conclusions

Conclusions and recommendations on cognitive testing after a TIA are hindered by significant heterogeneity between previous studies. Future research should focus on establishing a consensus on tools, definitions, and time-points, and validating tools specifically for the TIA population. Additionally, newer tests which are freely available, computerised, or designed specifically for vascular-type cognitive impairment should be evaluated.

## Supplementary Material

Supplemental Material

## Data Availability

It is confirmed by the authors that the data generated from this study are available both within the article as well as the accompanying supplementary material.

## Appendix 1. National institutes of health quality assessment scale

**Table:**

Criteria	Yes	No	Other (CD, NR, NA)*
1. Was the research question or objective in this paper clearly stated?			
2. Was the study population clearly specified and defined?			
3. Was the participation rate of eligible persons at least 50%?			
4. Were all the subjects selected or recruited from the same or similar populations (including the same time period)? Were inclusion and exclusion criteria for being in the study prespecified and applied uniformly to all participants?			
5. Was a sample size justification, power description, or variance and effect estimates provided?			
6. For the analyses in this paper, were the exposure(s) of interest measured prior to the outcome(s) being measured?			
7. Was the timeframe sufficient so that one could reasonably expect to see an association between exposure and outcome if it existed?			
8. For exposures that can vary in amount or level, did the study examine different levels of the exposure as related to the outcome (e.g., categories of exposure, or exposure measured as continuous variable)?			
9. Were the exposure measures (independent variables) clearly defined, valid, reliable, and implemented consistently across all study participants?			
10. Was the exposure(s) assessed more than once over time?			
11. Were the outcome measures (dependent variables) clearly defined, valid, reliable, and implemented consistently across all study participants?			
12. Were the outcome assessors blinded to the exposure status of participants?			
13. Was loss to follow-up after baseline 20% or less?			
14. Were key potential confounding variables measured and adjusted statistically for their impact on the relationship between exposure(s) and outcome(s)?			
