# Supplementary material for: Cognitive testing following transient ischaemic attack: A systematic review of clinical assessment tools
Source: Cogent Psychol. 2023 Apr 1;10(1):2196005. doi: 10.1080/23311908.2023.2196005 (PMC10069374; doi:10.1080/23311908.2023.2196005)
Supplement: Supplemental Material [file OAPS_A_2196005_SM7381.docx]

**Supplementary material 1 (S1)**

**Search criteria**

1. TIA

2. Transient Ischemic attack*

3. Transient ischaemic attack*

4. 2 OR 3

5. Minor stroke

6. Mini stroke

7. Mild stroke

8. Non-disabling stroke

9. Nondisabling stroke

10. RIND

11. Reversible ischemic neurological deficit

12. Reversible ischaemic neurological deficit

13. 1 OR 4 OR 5 OR 6 OR 7 OR 8OR 9 OR 10 OR 11 OR 12

14. Cogniti*

15. Dement*

16. Memory

17. Recall

18. “executive function”

19. Planning

20. Reasoning

21. Language

22. Attention

23. Orientation

24. 14 OR 15 OR 16 OR 17 OR 18 OR 19 OR 20 OR 21 OR 22 OR 23

25. Assess*

26. Screen*

27. Measure

28. Scale

29. Battery

30. Test

31. Tool

32. Index

33. 25 OR 26 OR 27 OR 28 OR 29 OR 30 OR 31 OR 32

34. 13 AND 24 AND 33

35. Accuracy

36. Validity

37. Specificity

38. Sensitivity

39. 35 OR 36 OR 37 OR 38

40. 34 AND 39
